# All-dielectric carpet cloaks with three-dimensional anisotropy control

**DOI:** 10.1515/nanoph-2022-0786

**Published:** 2023-06-14

**Authors:** Yuki Maegawa, Yosuke Nakata, Atsushi Sanada

**Affiliations:** Graduate School of Engineering Science, Osaka University, Toyonaka, Japan; Center for Quantum Information and Quantum Biology, Osaka University, Toyonaka, Japan

**Keywords:** anisotropy control, carpet cloaks, invisibility cloaks, metamaterials, transformation optics

## Abstract

In this article, we propose all-dielectric carpet cloaks composed of jungle gym shaped dielectric unit cells and present a design strategy for three-dimensional (3-D) anisotropy control based on the transformation optics. The carpet cloaks are 3-D printable and operate with polarization independent incident waves in arbitrary incident angles due to the 3-D anisotropy control. Realizable anisotropic permittivities of cubic and rectangular unit cells are numerically studied based on the relative permittivity and loss tangent of *ɛ*_r_ = 2.9 and tan *δ* = 0.02 of ultra-violet curing resin measured at the microwave frequency. It is shown that the unit cell has little frequency dependence even with the anisotropy in the low frequency range where the effective medium approximation is valid. A carpet cloak is designed based on the design method with a quasi-conformal coordinate transformation and implemented with the unit cells taking into account its realizable anisotropy. Polarization independent 3-D cloaking operations of the designed cloak are confirmed numerically. The designed cloak is fabricated by stereolithography 3-D printing technology and its cloaking performances are evaluated experimentally at 10 GHz. It is shown that non-specular reflections are well suppressed by the carpet cloak for both TE and TM incident waves with different incident angles of 30, 45, and 60°. Frequency independent cloaking operations are also shown experimentally in the X-band. The measured near-field distributions and bistatic radar cross sections are in good agreement with simulated predictions and the validity of the design method is confirmed.

## 1 Introduction

The carpet cloaks [1] are reflection type invisibility cloaks that eliminate non-specular scattered waves based on the transformation optics [2]. All-dielectric carpet cloak have been implemented at the optical frequencies [3–10] and at the microwave frequencies [11, 12] to drastically reduce losses compared with ones made with metal [13–17]. Isotropic effective refractive index control approaches have been taken in conventional all-dielectric carpet cloak designs; two-dimensional (2-D) carpet cloaks at the optical frequencies have been presented by drilling holes on a dielectric substrate with focused ion beam (FIB) [3, 4] or creating dielectric posts by electron-beam (EB) lithography [5] to control 2-D isotropic effective refractive index. Similar approach has also been taken at the microwave frequencies [18]. Polarization independent three-dimensional (3-D) carpet cloaks have been presented by woodpile photonic crystals fabricated by direct laser writing [6, 7] at the visible light frequencies. Inhomogeneous isotropic dielectric materials fabricated by drilling inhomogeneous holes in layered dielectric plates [11] have also been used to realize a 3-D carpet cloak at the microwave frequencies. However, all of these approaches are still within the category of the isotropic homogenization approach, which essentially yields polarization dependency and fundamentally degrades the polarization characteristics. General and practical design methods for 3-D anisotropy control have not been presented so far to the authors’ best knowledge.

On the other hand, uniaxial anisotropy control approaches have been introduced to realize carpet cloaks for easy design and fabrication; for instance, uniform natural uniaxial calcite crystal has been used to demonstrate the carpet cloaking at the optical frequencies [19–21]. Stratified dielectric media [22, 23] have also been introduced to control uniaxial anisotropy. Although the uniaxial anisotropy control approach is advantageous in terms of omitting position dependent anisotropy control, the approach is applicable only for certain isovolumetric coordinate transformations [19–24], which restricts the cloaking areas to rectilinear shapes and drastically limits the flexibility of cloaking applications. In order to use general coordinate transformations in the transformation optics, position dependent 3-D anisotropy control design strategies are required.

Three-dimensional printing technology is a much more flexible and inexpensive manufacturing scheme for metamaterial fabrication [25–35] including all-dielectric carpet cloaks [26, 33] compared with other fabrication technologies using conventional EB photolithography, FIB, two-photon polymerization [36], and the like. Although the 3-D printing technology is potentially capable of inhomogeneous 3-D anisotropy control, only isotropic carpet cloaks such as gradient index dielectric photonic crystals [26] or randomly patterned dielectric metamaterials [33] have been introduced with 3-D printing technology so far.

In this article, we propose all-dielectric carpet cloaks composed of jungle gym shaped dielectric unit cells and present a design strategy for 3-D anisotropy control based on the transformation optics. The carpet cloaks are 3-D printable and operate with polarization independent incident waves in arbitrary incident angles due to the 3-D anisotropy control. The effective permittivity and its anisotropy are studied numerically with measurement based realistic host dielectric’s material parameters obtained by the free-space method [37] at the microwave frequencies. The frequency dependence of the unit cell is also discussed. A carpet cloak is designed based on the proposed design method with a quasi-conformal coordinate transformation and implemented taking into account its realizable 3-D anisotropy of the unit cells. Numerical simulations are carried out to reveal the cloaking performances for TM and TE incident waves. The designed cloak is fabricated by stereolithography 3-D printing technology and its polarization independent cloaking performance is studied based on near-field measurements at 10 GHz with different incident angles of 0, 30, and 60°. Frequency dependencies of the cloak in the X-band are also discussed experimentally.

## 2 Carpet cloaks with jungle gym unit cells

**Figure 1:**
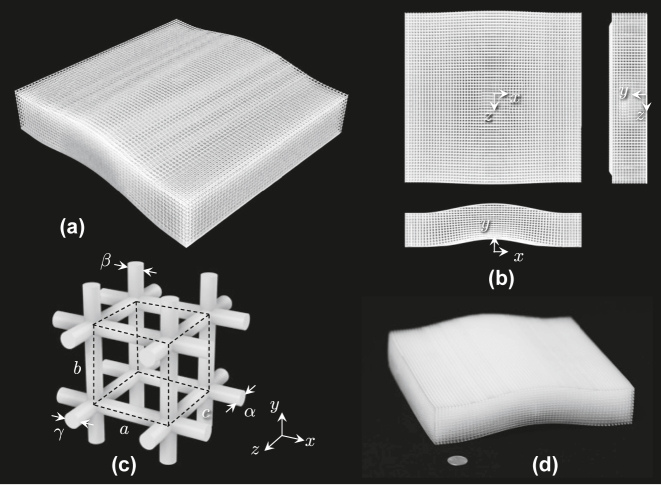
Proposed carpet cloak. (a) General view. (b) Three-side view. (c) Unit cell (shown with the dashed lines). (d) 3-D printed prototype. The total size of the prototype is 180 mm × 35 mm × 180 mm. The coin in the picture is a United States quarter.

Figure 1(a) and (b) show the proposed carpet cloak. The carpet cloak consists of jungle gym shaped unit cells whose sides are made of cylindrical dielectric bars as shown in Figure 1(c). The effective permittivity tensor components of the unit cell are controlled by the diameters of the cylindrical side bars, *α*, *β*, and *γ* in the *x*-, *y*-, and *z*-directions, respectively, as well as the lattice constants, *a*, *b*, and *c* in the *x*-, *y*-, and *z*-directions, respectively. The carpet cloak is 3-D printable and a prototype made of ultra-violet (UV) curing resin fabricated by the stereolithography 3-D printer is shown in Figure 1(d). Note that the effective permittivity is almost frequency independent and the cloak fundamentally operates in an extremely wide frequency range from DC to a certain frequency where the unit cell size is small enough compared with the wavelength, which will be shown in the following section.

## 3 Unit cell anisotropy

**Figure 2:**
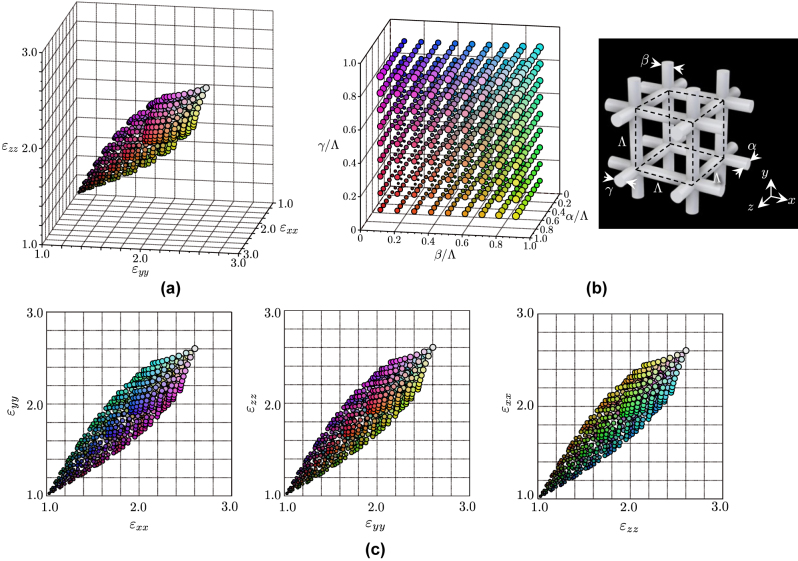
Simulated effective relative permittivities for a various combinations of the side bar diameters (*α*, *β*, and *γ*) of a cubic unit cell (*a* = *b* = *c*, *ɛ*_r_ = 2.9, and tan *δ* = 0.02). (a) Effective relative permittivity values plotted in the *ɛ*_*xx*_ *ɛ*_*yy*_ *ɛ*_*zz*_-space. The dot color corresponds to each structural parameter. (b) Dot color versus (*α*, *β*, and *γ*) diameter values. (c) Permittivity projection on the *ɛ*_*xx*_ *ɛ*_*yy*_-, *ɛ*_*yy*_ *ɛ*_*zz*_-, and *ɛ*_*zz*_ *ɛ*_*xx*_-planes.

In order to design the cloak, the effective anisotropic permittivity of the unit cell is studied. The relationship between a set of side bar diameter values (*α*, *β*, and *γ*) and a set of effective relative permittivity diagonal component values (*ɛ*_*xx*_, *ɛ*_*yy*_, and *ɛ*_*zz*_) are databased by using full-wave eigenmode simulations with the Bloch–Floquet periodic boundary conditions based on the finite element method. Material parameter measurements for a host dielectric of UV curing resin used for a stereolithography 3-D printer are carried out in advance by the free-space method [37] at 10 GHz, and the relative permittivity of *ɛ*_r_ = 2.9 and the loss tangent tan *δ* = 0.02 are used in the simulation according to the measured results. Figure 2(a) illustrates the calculated relative permittivity points (*ɛ*_*xx*_, *ɛ*_*yy*_, and *ɛ*_*zz*_) in the *ɛ*_*xx*_ *ɛ*_*yy*_ *ɛ*_*zz*_-space for a variety of combinations of the side bar diameter values (*α*, *β*, and *γ*) with the parameter range of 0.1Λ ≤ *α*, *β*, *γ* ≤ 0.9Λ for a cubic unit cell with *a* = *b* = *c* ≡ Λ. Each dot color corresponds to a set of the diameter values (*α*, *β*, and *γ*) plotted in Figure 2(b). Figure 2(c) shows the projections of Figure 2(a) on the *ɛ*_*xx*_ *ɛ*_*yy*_-, *ɛ*_*yy*_ *ɛ*_*zz*_-, and *ɛ*_*zz*_ *ɛ*_*xx*_-planes. For anisotropic permittivity values required in the cloak design, the structural parameters of the unit cell are determined based on the database with linear interpolations. In this case, the *ɛ*_*ii*_ value ranges from 1.0 to 2.8 within the range of each *α*, *β*, and *γ* value from 0.1Λ to 0.9Λ. Note that the realizable anisotropy delimits the maximum cloaking area. For instance, the ratio of the maximum and minimum *ɛ*_*ii*_ values, *ɛ*_*ii*, max_/*ɛ*_*jj*, min_, is 1.2 in this case with (*α*, *β*, and *γ*) = (0.1Λ, 0.6Λ, and 0.7Λ) (or symmetric combinations, e.g. (0.1Λ, 0.6Λ, and 0.7Λ) and the like). Note that this value depends on the permittivity value of the host medium. The larger the permittivity of the host medium, the larger the anisotropy.

**Figure 3:**
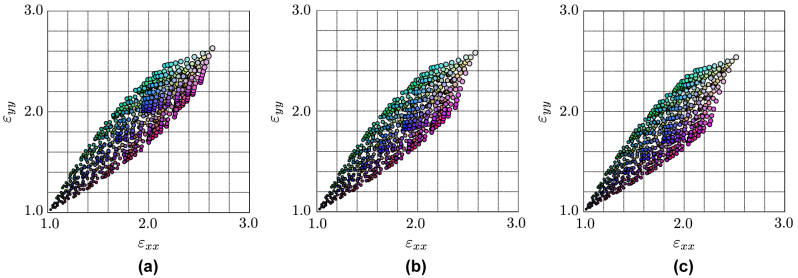
Simulated effective relative permittivities for rectangular unit cells for (a) *b* = 0.95Λ, (b) *b* = 1.05Λ, and (c) *b* = 1.15Λ with various combinations of the side bar diameters (*α*, *β*, and *γ*), where *a* = *c* = Λ. The colored dots indicate structural parameters shown in Figure 2(b). Plots only on the *ɛ*_*xx*_ *ɛ*_*yy*_-plane are shown.

Rectangular unit cells derived from the quasi-conformal coordinate transformation are also used in the cloak design, which also affects the permittivity anisotropy. Figure 3 shows examples of simulated permittivity tensor diagonal component plots for rectangular unit cells with *a* = *c* = Λ and *b* ≠ Λ. Figure 3(a)–(c) are for the cases with *b* = 0.95Λ, 1.05Λ, and 1.15Λ, respectively, which covers the anisotropy range from 0.97 ≤ *ɛ*_*ii*, max_/*ɛ*_*jj*, min_ ≤ 1.13 discussed later in the cloak design. The colored dots indicate structural parameters shown in Figure 2(b). The permittivity and the loss tangent of *ɛ*_r_ = 2.9 and tan *δ* = 0.02 are used in the simulations. As seen in the figure, it is intuitive that the anisotropy becomes larger as one of the lattice sides *b* changes. In the case with *b* = 1.15Λ, the maximum ratio among the tensor component values *ɛ*_*ii*, max_/*ɛ*_*jj*, min_ slightly increases from 1.2 of the cubic case to 1.22 with (*α*, *β*, and *γ*) = (0.7Λ, 0.1Λ, and 0.8Λ) and (0.8Λ, 0.1Λ, and 0.7Λ).

**Figure 4:**
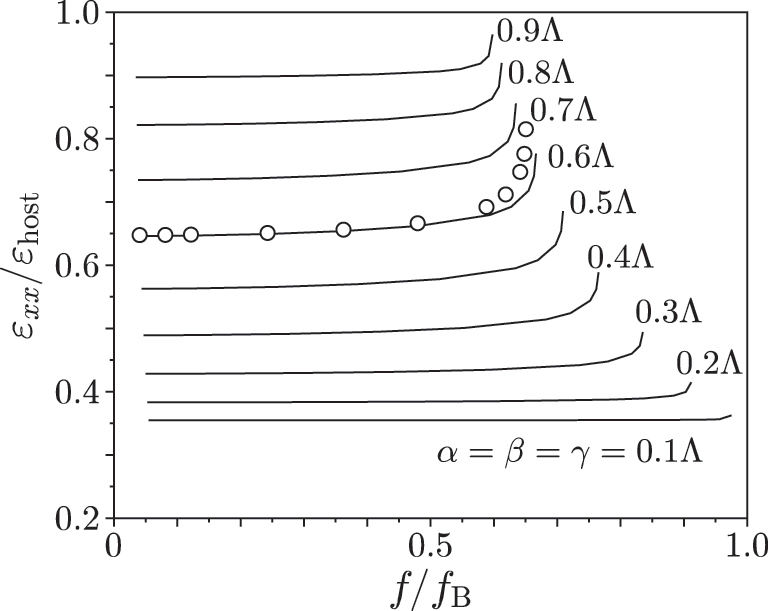
Frequency dependence of a permittivity tensor component. Lines are for cubic unit cells with *α* = *β* = *γ* from 0.1Λ to 0.9Λ in 0.1Λ increments. Circles are for the case with (*α*, *β*, and *γ*) = (0.1Λ, 0.6Λ, and 0.7Λ) where the anisotropy is strongest.

Figure 4 shows an example of the frequency dependence of the *ɛ*_*xx*_ (= *ɛ*_*zz*_ = *ɛ*_*zz*_) for a cubic unit cell (*a* = *b* = *c* = Λ). The horizontal axis indicates the normalized frequency by the Bragg frequency *f*_B_ = *c*_0_/2Λ, and the vertical axis indicates the relative permittivity *ɛ*_*xx*_ normalized by that of the host media (*ɛ*_host_ = 2.9). Lines are for the cases with the side bar diameters *α* = *β* = *γ* from 0.1Λ to 0.9Λ. It is seen from the figure that *ɛ*_*xx*_ is almost frequency independent in the low frequency band where *f*/*f*_B_ is small enough and the effective medium approximation is valid. For instance, in the range where *f*/*f*_B_ < 0.1 (the unit cell size is smaller than *λ*_0_/10, where *λ*_0_ is the wavelength in free space), *ɛ*_*xx*_ can be considered to be frequency independent regardless of the bar diameters, which suggests ultra-broadband cloaking operation. The permittivity increases rapidly near *f*_B_ due to the slow wave effect. Note that the singular frequency shifts with the diameter values *α* = *β* = *γ* toward the lower frequency since the effective wavelength decreases and the effective Bragg frequency decreases with the increasing diameter values *α* = *β* = *γ*. It is also seen from the figure that the frequency dependence becomes slightly large if the diameters *α* = *β* = *γ* ∼ 0.5Λ compared with the cases with *α* = *β* = *γ* close to 0 or Λ.

Circles in Figure 4 show the effective permittivity *ɛ*_*xx*_ for the case with (*α*, *β*, *γ*) = (0.1Λ, 0.6Λ, and 0.7Λ) where the anisotropy is strongest. As seen from the figure, the frequency dependency is still small in the low frequency band even with the strongest anisotropy.

## 4 Design

### 4.1 Coordinate system and material parameters

**Figure 5:**
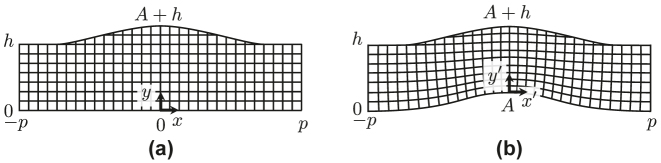
Original and transformed areas and the coordinate systems. (a) Original area. (b) Transformed area.

Here, we consider the coordinate transformation in the *xy*-plane from the original area of Figure 5(a) bounded by the three lines and the quartic function given by

(1) $$\begin{array}{cc} x=\pm \frac{w}{2},\;\;y & =0,\;and\;\\ y & =\left\{\begin{array}{cc} h+A{\left[1-{\left(\frac{x}{p}\right)}^{2}\right]}^{2}\; & \left(|x|\leq p\right)\\ h\; & \left(|x|>p\right) \end{array}\right. \end{array}$$

into the area of Figure 2(b) whose bottom shape is identical to that of the top boundary, i.e.,

(2) $$\begin{array}{cc} x=\pm \frac{w}{2},\;\;y & =\left\{\begin{array}{cc} A{\left[1-{\left(\frac{x}{p}\right)}^{2}\right]}^{2}\; & \left(|x|\leq p\right)\\ 0\; & \left(|x|>p\right) \end{array}\right.,\;and\;\;\\ y & =\left\{\begin{array}{cc} h+A{\left[1-{\left(\frac{x}{p}\right)}^{2}\right]}^{2}\; & \left(|x|\leq p\right)\\ h\; & \left(|x|>p\right) \end{array}\right.. \end{array}$$

where *h* and 2*w* are the height and the width of the carpet, respectively, and *A* and 2*p* are the height and the width of the cloaking area, respectively. The cloak is assumed to be uniform in the *z*-direction. A coordinate system in the transformed area is determined numerically, and nominal effective permittivity and permeability tensor parameters are obtained based on the transformation optics theory [2] as

(3) $$\varepsilon_{ij}=\mu_{ij}=\sqrt{g}g_{ij}$$

where *g*_*ij*_ is the metric tensor of the transformed coordinate system in Figure 5(b).

For practical implementations of all-dielectric cloaks with the 3-D anisotropy control, we need to deal with the physical constraints where (A) the permittivity tensor components including off-diagonal components have to be controlled individually for both the TE and TM incident waves, (B) the permeability tensor has to be unitary, and (C) all the permittivity tensor components have to be larger than unity since we target only non-resonant implementations. In order to overcome these constraints, we develop a design strategy as follows: To overcome Constraint A, first, we use a quasi-conformal transformation to essentially suppress the off-diagonal components of the permittivity and permeability tensors. Then, we calculate the permittivity and permeability tensors according to (3) and simply neglect the component values. In addition, to overcome Constraint B, we deform the tensor parameters at a cost of small reflections by letting the diagonal components of the permeability tensor be unity conserving effective refractive indices for different polarizations individually; in concrete, for TM incident waves whose polarization is in the *xy*-plane in the coordinate system in Figure 5, we multiply both *ɛ*_*xx*_ and *ɛ*_*yy*_ by *μ*_*zz*_. On the other hand, for *z*-polarized TE incident waves, we multiply *ɛ*_*zz*_ by the average of the *μ*_*zz*_ and *μ*_*yy*_ values. Finally, in order to overcome Constraint C, we divide both the *xx*- and *yy*-components of the permittivity tensor by the minimum value in *ɛ*_*xx*_ and *ɛ*_*yy*_, namely *ɛ*_TM,min_, for the TM incident waves. Similarly, we divide the *zz*-component by the minimum value of *ɛ*_*zz*_, namely *ɛ*_TE,min_, for TE incident waves. Eventually, we end up with the permittivity and permeability tensors for the implementation as

(4) $$\begin{array}{cc} {\bar{\bar{\varepsilon }}}^{\prime } & =\left(\begin{array}{ccc} \frac{\varepsilon_{xx}\mu_{zz}}{\varepsilon_{\text{TM,min}}} & 0 & 0\\ 0 & \frac{\varepsilon_{yy}\mu_{zz}}{\varepsilon_{\text{TM,min}}} & 0\\ 0 & 0 & \frac{\varepsilon_{zz}}{\varepsilon_{\text{TE,min}}}\frac{\mu_{xx}+\mu_{yy}}{2} \end{array}\right),\;\;\\ {\bar{\bar{\mu }}}^{\prime } & =\left(\begin{array}{ccc} 1 & 0 & 0\\ 0 & 1 & 0\\ 0 & 0 & 1 \end{array}\right). \end{array}$$

**Figure 6:**
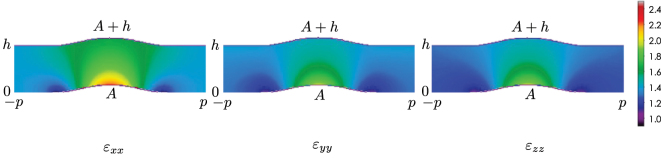
Diagonal relative permittivity tensor components for the carpet cloak. The off-diagonal components are assumed to be zero.

The cloak dimensions of *A* = *λ*_0_/3, *p* = 2*λ*_0_, *h* = 7*λ*_0_/6, *w* = 5*λ*_0_/2 are chosen in this implementation considering the realizable anisotropy of the host dielectric material with *ɛ*_r_ = 2.9, where *λ*_0_ is the wavelength at the design frequency *f*_0_. A transformed coordinate system is numerically determined based on the quasi-conformal coordinate transformation, and the permittivity tensor parameters are calculated according to the procedure shown in the previous section. Figure 6 shows the obtained diagonal components of the permittivity tensor given by (4). The maximum off-diagonal component value is less than 0.03 in this quasi-conformal coordinate transformation and the off-diagonal components are ignored.

Now, we will discuss the effect of the deformations on cloak performances. For the deformation of neglecting the off-diagonal components due to Constraint A, the maximum off-diagonal component values is 0.03 thanks to the quasi-conformal transformation [1], and there will be little effect on the cloaking performance. For the deformations due to Constraints B and C, the reflections occur at the interface due to the impedance mismatch. According to the permittivity tensor component values of Figure 6, the *ɛ*_*xx*_ has the maximum value of 1.6 on the top interface, which leads to the reflection coefficient of $|\left(\sqrt{1/\varepsilon_{xx}}-1\right)/\left(\sqrt{1/\varepsilon_{xx}}+1\right)|=0.12$ . The other components, *ɛ*_*yy*_ and *ɛ*_*zz*_, have permittivity values less than 1.3 at most on the interface, leading to the reflection coefficients less than 0.07. These may degrade the cloaking performance for some incident angles with specific polarizations.

### 4.2 Structural parameters

**Figure 7:**
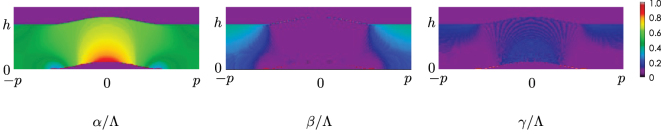
Designed bar diameters.

The structural parameters of the carpet cloak having the designed permittivity tensor parameters of Figure 6 are designed. The diameters of the cylindrical dielectric bars in the unit cell, *α*, *β*, and *γ*, are discretized each from 0.1Λ to 0.9Λ, in 0.1Λ increments for the cases with the lattice constant parameter defined by *ζ* ≡ *b*/Λ $\left(≃\sqrt{g_{xx}}/\sqrt{g_{yy}}\right)$ from 0.95 to 1.25, in 0.05 increments and the diagonal permittivity tensor parameters are full-wave simulated and databased. These structural parameters of the unit cells are determined based on the database so that the effective permittivity tensor parameters can simultaneously satisfy the designed permittivity tensor components shown in Figure 6 with linear interpolations. Figure 7 shows the bar diameters of the unit cell (*α*, *β*, and *γ*) at the sample points of the unit cell centers obtained from the database. Note that not all the unit cells satisfy the parameters due to the lack of the anisotropy range; in this design, the ratios of the number of the unit cells with the error less than 0.01 % from the theoretical value to the total number of unit cells are 82.4 %, 95.5 % and 92.8 % for *ɛ*_*xx*_, *ɛ*_*yy*_, and *ɛ*_*zz*_, respectively. The maximum errors of the *ɛ*_*xx*_, *ɛ*_*yy*_, and *ɛ*_*zz*_ values are 0.07, 0.15, and 0.18, respectively, which potentially cause additional scatterings in cloaking operations.

## 5 Cloaking performance simulations

### 5.1 Scattering characteristics

In order to confirm the scattering suppression performances of the designed cloak, numerical simulations are carried out at 10 GHz based on the full-tensor anisotropic circuit model [38]. The carpet cloak with the designed parameters of (4) is located at the bottom of a computational area with 480 × 480 unit cells whose sides are *λ*_0_/10. The bottom boundary of the cloak is short-circuited for TE incident wave or open-circuited for TM incident wave to generate perfect reflections. The cloak is illuminated by continuous wave sources having the internal impedance of the free-space impedance *η*_0_ = 377 Ω with the aperture size of 2.33 *λ*_0_. The periphery of the computational area is terminated by the free-space impedance *η*_0_ to prevent reflections.

**Figure 8:**
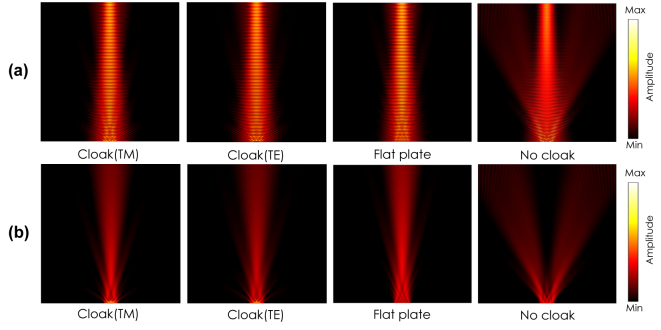
Scattering characteristics (simulation). *θ*_*i*_ = 0°. (a) Total electric field distributions. (b) Scattered field distributions.

Figure 8 shows the simulated field distributions with the incident angle of *θ*_*i*_ = 0° for (a) the total incident and scattered field and (b) the scattered field extracted from the total field by subtracting the incident field calculated separately. The incident angle *θ*_*i*_ is the angle from the *y*-axis in the *xy*-plane (see the inset in Figure 13). The figures from left to right show the results for the cases with the cloak illuminated by the TM incident wave, the cloak illuminated by the TE incident wave, for a flat plate, and for a bump without the cloak, respectively. It is clearly seen from the figures that the incident scatted waves are reflected back to the normal directions for both polarizations as seen in the case of the flat plate, whereas the incident wave is scattered in oblique directions by the bump.

**Figure 9: j_nanoph-2022-0786_fig_009:**
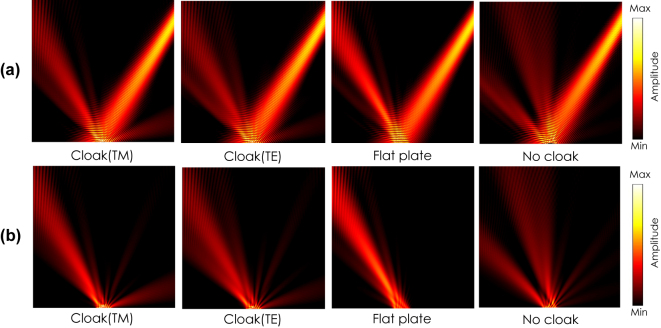
Scattering characteristics (simulation). *θ*_*i*_ = 30°. (a) Total electric field distributions. (b) Scattered field distributions.

**Figure 10:**
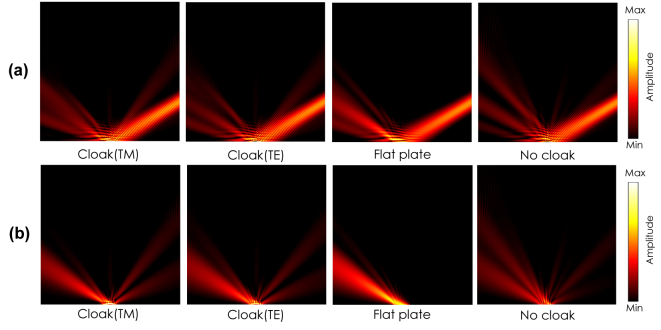
Scattering characteristics (simulation). *θ*_*i*_ = 60°. (a) Total electric field distributions. (b) Scattered field distributions.

Figures 9 and 10 show the similar results with the incident angles of *θ*_*i*_ = 30° and 60°, respectively. It is seen from these figures that the TM incident wave is mostly reflected to the specular direction, however the TE incident wave is scattered to some extent compared with the TM incident wave case. The performance difference is due to the difference in the material parameter approximations without magnetic control; i.e., for the TE incident wave case, the cloaking operation relies on only *ɛ*_*zz*_, whereas for the TM incident wave case, the operation relies on both *ɛ*_*xx*_ and *ɛ*_*yy*_. It is also seen in 9(b) and 10(b) that backward scatterings are observed under larger angle incidences both TE and TM waves. The root cause is not identified yet; however possible causes are due to the parameter deformations given in Section 4.1 as well as discretization errors.

**Figure 11:**
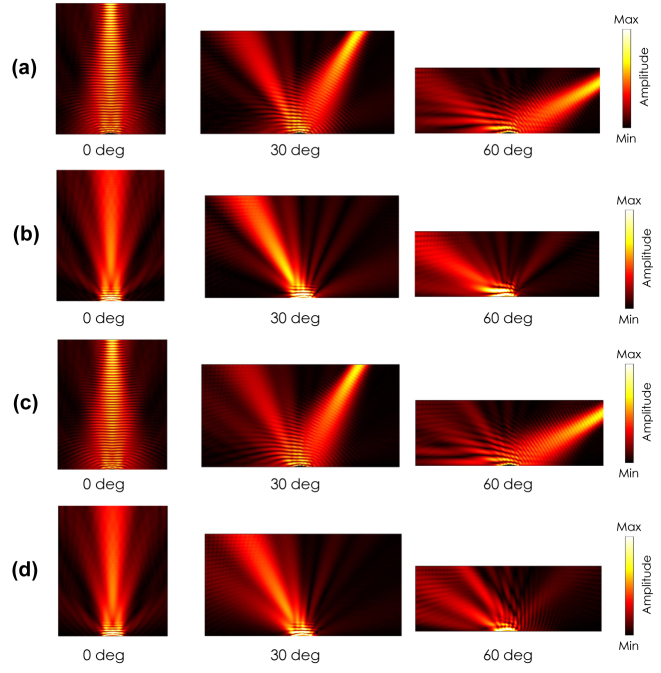
Comparison of a cloaking performance between the proposed anisotropic cloak and a conventional isotropic cloak with the TM incident waves with the angles of 0, 30, and 60° (simulation). (a) Total and (b) scattered field distributions for the proposed carpet cloak. (c) Total and (d) scattered field distributions for the isotropic carpet cloak.

Figure 11 shows a performance comparison between the proposed anisotropic cloak and a conventional isotropic cloak [1]. The isotropic cloak is designed based on an effective dielectric medium with the averaged refractive index for the TM incident wave; i.e., $n=\sqrt{\mu_{xx}\mu_{yy}}/\sqrt{\varepsilon_{zz}}$ and *μ* = 1. The quasi-conformal transformation is also used to minimize the off-diagonal components. The computational regions with 300 × 360, 500 × 260, and 480 × 170 unit cells are prepared for an incident wave with the angles of *θ*_*i*_ = 0, 30, and 60°, respectively. The unit cell size is *λ*_0_/10 × *λ*_0_/10. Figure 11(a) and (b) show the simulated total and scattered field distributions for the proposed anisotropic cloak, respectively, and Figure 11(c) and (d) show corresponding simulated total and scattered field distributions for the isotropic cloak, respectively. It is seen from the figures that the reflected waves are directed in the specular angles of 0, 30, and 60° for the anisotropic cloak as seen in Figure 11(a) and (b), whereas the reflected waves are scattered and the cloak performance deteriorates as the incident angle becomes large for the conventional isotropic cloak as seen in Figure 11(c) and (d). From these results, it is concluded that the separate control of *ɛ*_*xx*_ and *ɛ*_*yy*_ contributes to preventing cloak performance deteriorations with large incident angles, which is an advantage of the proposed anisotropic cloak.

### 5.2 Bistatic radar cross sections

**Figure 12:**
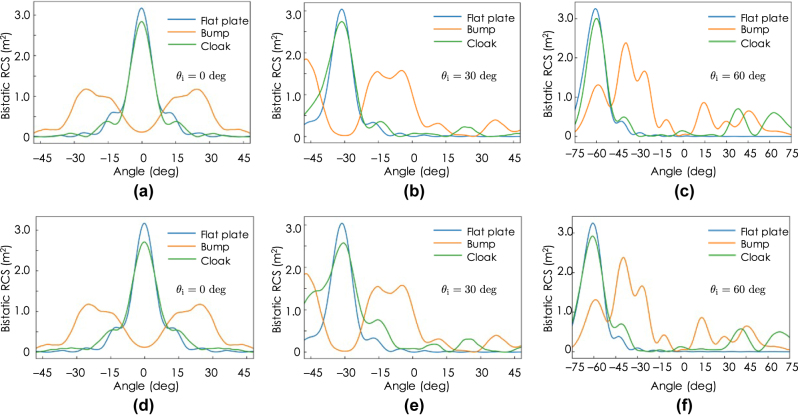
Bistatic radar cross sections (simulation). For the TM incident wave with (a) *θ*_*i*_ = 0°, (b) *θ*_*i*_ = 30°, and (c) *θ*_*i*_ = 60°. For the TE incident wave with (a) *θ*_*i*_ = 0°, (b) *θ*_*i*_ = 30°, and (c) *θ*_*i*_ = 60°.

In order to quantitatively evaluate the scattering suppression performance of the cloak, the bistatic radar cross sections (BRCSs) are calculated for the three cases of *θ*_*i*_ = 0, 30, and 60°. Figure 12(a)–(c) show BRCSs for the TM incident waves with the incident angles of *θ*_*i*_ = 0, 30, and 60°, respectively. For the normal incident case of (a), the reflected wave by the cloak is directed in the specular normal direction with little extra reflections, as opposed to the case by the bump where the incident wave is scattered in the directions approximately 10° ≤|θ|≤35 °. For the oblique incident cases of *θ*_*i*_ = 30 and 60° in (b) and (c), major reflections are directed in the specular directions with the cloak, whereas there is little reflections in the specular angles without the cloak. Note that there are small reflections in the positive directions; e.g., in the +25° direction for *θ*_*i*_ = 30° case and in the +35 and 65° directions for the case of *θ*_*i*_ = 60, which is considered to be due to imperfect implementations of the unit cells design as well as the theoretical approximations assumed in the permittivity tensor parameters.

Figure 12(e)–(f) shows similar BRCS calculation results for the TE incident waves with the incident angles of *θ*_*i*_ = 0, 30, and 60°, respectively. For the normal incident wave case of (d), most of the reflected power is reflected in the normal directions with little extra reflections in the case with the cloak. In addition, for the oblique incident cases of *θ*_*i*_ = 30 and 60°, the major reflections direct in the specular directions, however there are small reflections in the positive directions. This is also considered to be due to imperfect implementations of the unit cells as well as the theoretical approximations assumed in the permittivity tensor parameters.

## 6 Experiments

### 6.1 Prototype

The carpet cloak designed in Section 4 is fabricated by a stereolithography 3-D printer. The prototype is shown in Figure 2(d). The design frequency is chosen as *f*_0_ = 10 GHz. The nominal unit cell size is chosen to be 3 mm × 3 mm × 3 mm (=*λ*_0_/10 × *λ*_0_/10 × *λ*_0_/10) and the size is varied according to the designed coordinate system. Measured permittivity and loss tangent of the UV curing resin used by the 3-D printer are *ɛ*_r_ = 2.9 and tan *δ* = 0.02. The nominal resolution of the 3-D printer is 50 μm and the maximum fabrication error range estimated by point samplings is ±75 μm. The total size of the prototype is 180 mm × 35 mm × 180 mm. The prototype is backed by a copper tape without any gaps. For comparison, a flat plate with the same footprint of the cloak and a bump with the same bottom shape of the cloak are fabricated by the 3-D printer. The flat plate and the bump are covered by the copper tape on the surface.

### 6.2 Measurement system

**Figure 13:**
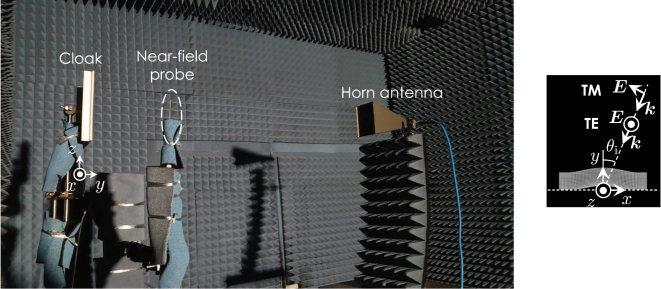
Measurement system.

Figure 13 shows the measurement system for the scattering characteristics. The prototype is mounted on a sample fixture in an anechoic chamber with the orientation shown in the figure. The prototype is illuminated with a standard 23 dB X-band horn antenna positioned for specific incident angles of 0°, 30°, and 60°. The incident wave polarization is in either horizontal or vertical directions depending on the horn antenna roll angle. The scattered fields in front of the prototype are picked up by an electric field probe and the amplitude and phase of a local electric field are measured with a vector network analyzer. The probe is scanned by an automated *xyz*-stage with the sampling period of 7.5 mm (=*λ*_0_/4) in the measurement areas of 480 mm × 480 mm, 480 mm × 480 mm, and 480 mm × 240 mm in the *xy*-plane for the cases with the incident angles of *θ*_*i*_ = 0°, 30°, and 60°, respectively. For comparison, similar measurements are carried out with the flat plate and the bump.

### 6.3 Near-field measurements

#### 6.3.1 TM incidence

**Figure 14:**
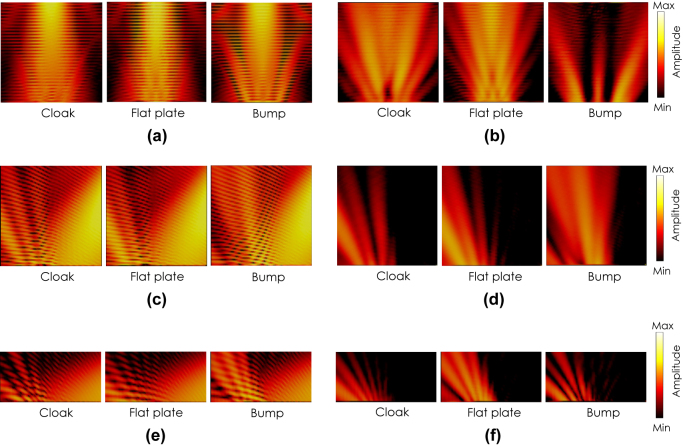
Measured field distributions for a TM incident wave. (a) Total and (b) scattered electric field distributions with *θ*_*i*_ = 0°. (c) Total and (d) scattered electric field distributions with *θ*_*i*_ = 30°. (e) Total and (f) scattered electric field distributions with *θ*_*i*_ = 60°.

Figure 14 shows the measured electric field distributions for the TM incident wave polarized in the *x*-direction with the incident angle *θ*_*i*_ = 0° for the cases with the cloak, the flat plate and the bump without the cloak. Figure 14(a) shows the total electric field distributions and Figure 14(b) shows the scattered electric field distributions extracted by subtracting a separately measured electric field distribution without the prototype from the total field distribution. Note that only a single component (horizontal component in Figure 13) is measured by a small dipole probe in this case. It is seen from the figure that the cloak reflects the incident wave back to the normal direction for the most part as in the flat plate with slight scatterings in the directions of ±15°. On the other hand, the bump scatters the incident wave strongly in the directions of ±30° with a small specular reflection.

Figure 14(c) and (d) show the measured total and scattered field distributions for the TM incident wave polarized in the horizontal direction with the incident angles of *θ*_*i*_ = 30°, respectively. Also, Figure 14(e) and (f) show the measured total and scattered field distributions for the TM incident wave polarized in the horizontal direction with the incident angles of *θ*_*i*_ = 60°, respectively. In each figure, measured field distributions with the cloak, the flat plate and the bump without the cloak are shown from the left to the right. It is seen from these figures that the cloak reflects the incident wave in the specular direction for the most part with small parasitic scatterings, which is qualitatively agree with those with the flat plate. On the other hand, the fields by the bump with strong scatterings in other directions with much less specular reflection. The difference between the measured field distributions and the simulated ones in Figures 8 [Fig j_nanoph-2022-0786_fig_009]–10 are considered to be due to errors in fabrication, material parameter measurement, and near-field measurements.

#### 6.3.2 TE incidence

**Figure 15:**
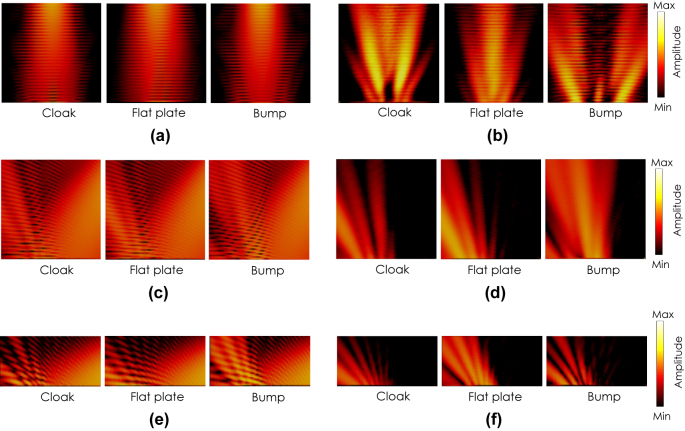
Measured field distributions for a TE incident wave. (a) Total and (b) scattered electric field distributions with *θ*_*i*_ = 0°. (c) Total and (d) scattered electric field distributions with *θ*_*i*_ = 30°. (e) Total and (f) scattered electric field distributions with *θ*_*i*_ = 60°.

Figure 15 shows the measured electric field distributions for the TE incident wave polarized in the vertical direction with the incident angle *θ*_*i*_ = 0° for the cases with the cloak, the flat plate and the bump without the cloak. Figure 15(a) shows the total electric field distributions and Figure 15(b) shows the scattered electric field distributions extracted by the same method as that of the TM case. The *z*-component (vertical component in Figure 13) is measured by a small monopole probe in this case. It is seen from the figure that the cloak reflects the incident wave back to the normal direction for the most part as in the flat plate with slight scatterings in the directions of ±15°. On the other hand, the bump scatters the incident wave strongly in the direction of ±30° with a small specular reflection.

Figure 15(c) and (d) show the measured total and scattered field distributions for the TE incident wave polarized in the vertical direction with the incident angles of *θ*_*i*_ = 30°. Also, Figure 15(e) and (f) show the measured total and scattered field distributions for the TE incident wave polarized in the vertical direction with the incident angles of *θ*_*i*_ = 60°. In each figure, measured field distributions with the cloak, the flat plate and the bump without the cloak are shown from the left to the right. Also in this TM incident case, it is seen from these figures that the cloak reflects the incident wave in the specular direction for the most part with small parasitic scatterings, which is qualitatively agree with those with the flat plate. On the other hand, the fields by the bump with a strong scattering in other directions with much less specular reflection.

#### 6.3.3 Frequency dependencies

**Figure 16:**
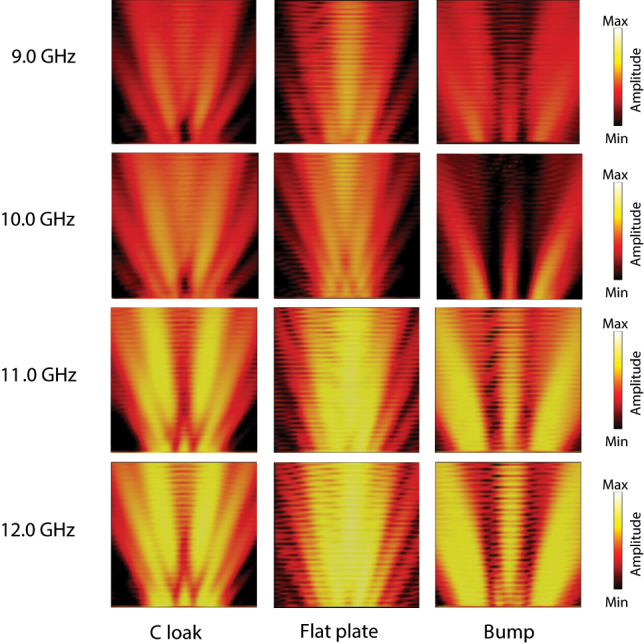
Measured frequency dependencies of scattered field distributions for a TM incident wave at 9.0, 10.0, 11.0, and 12.0 GHz. The incident angle is *θ*_*i*_ = 0°.

As suggested in Section 3, the jungle gym unit cell anisotropy has little frequency dependencies and broadband operations of the proposed cloak are expected as long as the wavelength is much larger than the unit cell size. Here, we see how the cloak behaves at different frequencies experimentally. Figure 16 shows scattered field distributions for a normal TM incident wave at 9.0, 10.0, 11.0, and 12.0 GHz in the X-band used in the experiments. It is seen from the figure that the cloak operation does not change much in the frequency band and the cloak suppresses scattered waves by the bump and directs the waves to the specular direction, which also suggests broadband operations of the proposed cloak experimentally. Note the unit cell sizes at 8.0 GHz and 12.0 GHz are 0.08*λ*_0,8GHz_ and 0.12*λ*_0,12GHz_, respectively. Here, *λ*_0,8GHz_ and *λ*_0,12GHz_ are the wavelengths at 8.0 GHz and 12.0 GHz, respectively. Incidentally, the intensity increment in the measured electric fields as the frequency increases in the figure is due to the sensitivity increment of the electric probe used in the measurement.

### 6.4 Bistatic radar cross sections

**Figure 17:**
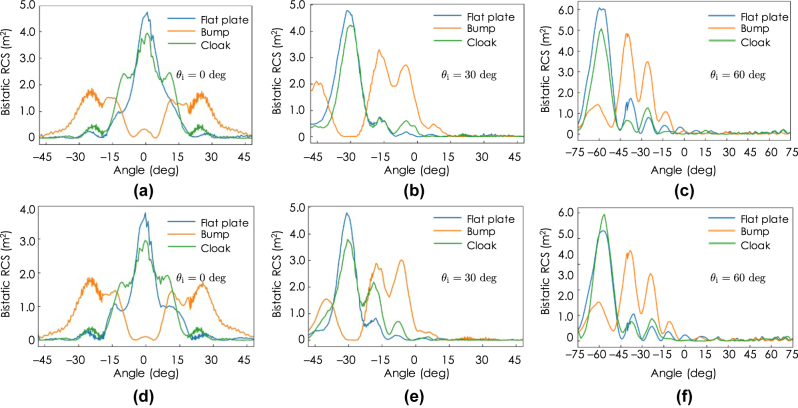
Measured bistatic radar cross sections. For the TM incident wave with (a) *θ*_*i*_ = 0°, (b) *θ*_*i*_ = 30°, and (c) *θ*_*i*_ = 60°. For the TE incident wave with (d) *θ*_*i*_ = 0°, (e) *θ*_*i*_ = 30°, and (f) *θ*_*i*_ = 60°.

In order to quantitatively evaluate the measured scattering characteristics, the BRCSs are calculated from the measured field distributions in Figures 14 and 15. Figure 17 shows the calculated BRCSs. Figure 17(a) and (d) are the BRCSs with the TM and TE incident waves, respectively, in the angle of *θ*_*i*_ = 0°. It is seen from these figures that the major part of the incident wave is reflected back to the normal directions without major peaks in the other directions except for larger peaks in approximately ±10° compared with those with the flat plate both for the TM and TE incident cases. This deterioration is not seen in the numerical predictions in Figure 12(a) and (d). Therefore, the cause of the performance deterioration is considered to be mainly due to the small number of imperfect unit cells that has errors in the effective permittivity parameters owing to the lack of the anisotropy range as well as errors in the fabrication and the measurements including the host material parameter measurements. On the other hand, in the cases with the bump, the scattered waves spread in the ranges from around −30° to −10° and from around 10° to 30° and have much less specular reflections both in the TM and TE incidence cases, which also agrees well with the numerical predictions in Figure 12(a) and (d).

Figure 17(b) and (e) are the BRCSs with the TM and TE incident waves, respectively, in the oblique angle of *θ*_*i*_ = 30°. For the TM incidence case of Figure 17(b), the BRCS with the cloak agree well with that with the flat plate except for minor peaks around −5° and the cloak reflects the incident wave to the specular direction of −30°, which is not the case with the bump; the BRCS with the bump has no peak in the specular direction of −30° and has major scattering peaks in the directions of around −5°, −20°, and −45°. This result qualitatively agrees well with the numerical prediction in Figure 12(b) in the angle range of *θ* < 15°, whereas there is much less reflection in the angle range of *θ* ≤ 15°. For the TE incidence case of Figure 17(e), the cloak reflects the major part of the incident wave in the specular direction of −30°, however the spurious levels in the −10° and −20° are relatively higher than those with the flat plate. The spurious levels are relatively higher than those in the TM incident case of Figure 17(b), which might be due to the fact that only *ɛ*_*zz*_-component is controlled in the TE incident case as opposed to the TM incident case with doubled *ɛ*_*xx*_- and *ɛ*_*yy*_-components control in this design. As for the bump case, there is no peak in the specular direction of −30° and has major scattering peaks in the non-specular directions of around −7°, −20°, and −40°, which also agrees well with the numerical predictions in Figure 12(b) and (e).

Figure 17(c) and (f) are the BRCSs with the angle of *θ*_*i*_ = 60° for the TM and TE incident waves, respectively. In these cases, the cloak reflects both the oblique TM and TE incident waves in the specular directions, which agree well with the case with the flat plate. On the other hand, for the cases with the bump, the major peaks are in the non-specular directions of approximately −40° and −25° with other small minor scatterings both for the TM and TE incidence cases. These results also qualitatively agree well with the numerical prediction in Figure 12(c) and (f).

## 7 Conclusions

We have presented all-dielectric carpet cloaks composed of jungle gym shaped dielectric unit cells and shown the design strategy for the 3-D anisotropy control of the transformation media. This approach not only improves polarization independent cloaking performances but also pave the way for general design and implementation schemes based on the transformation optics. The anisotropic properties of the jungle gym unit cell have been studied numerically and intrinsic broadband operations have been shown even with the anisotropy. A carpet cloak has been designed based on the presented design scheme and implemented by stereolithography 3-D printing technology with UV curing resin. Polarization independent 3-D cloaking operations of the designed cloak have been confirmed both numerically and experimentally. The prototype exhibits polarization and incident angle independent 3-D cloaking performances with broadband operations which agree well with the numerical predictions in terms of near-field distributions and BRCSs and the validity of the design method has been confirmed.
